# 3D printed scaffolds: Challenges toward developing relevant cellular *in vitro* models

**DOI:** 10.1016/j.bbiosy.2022.100044

**Published:** 2022-03-05

**Authors:** Beatriz Molina-Martínez, Luis M. Liz-Marzán

**Affiliations:** a CIC biomaGUNE, Basque Research and Technology Alliance (BRTA), 20014 Donostia-San Sebastián, Spain; b Biomedical Research Networking Center in Bioengineering, Biomaterials, and Nanomedicine (CIBER- BBN), 20014 Donostia-San Sebastián, Spain; c Ikerbasque, Basque Foundation for Science, 48009 Bilbao, Spain

**Keywords:** 3D Printing, Cell culture, Scaffolds

## Abstract

Three-dimensional (3D) cell culture technology has rapidly emerged, as a result of the increasing demand for improved *in vitro* systems that better resemble human physiology. Promising microphysiological systems have been fabricated by combining complex 3D culture with 3D-printing technologies. These models overperform existing *in vitro* systems regarding potential for biofabrication and predictive power. However, most systems under development do not ultimately find a long-term application. We provide herein an overview of the challenges to be considered when developing 3D *in vitro* systems by means of printed scaffolds, as well as some of the limitations of existing models.

## Highlights

• The use of 3D-printed technologies for the development of *in vitro* models requires consideration of multiple factors.
• The main objective is providing high reproducibility and reliability.
• Adequate printed scaffolds, stable cell growth, constant perfusion techniques, and reduction of 3D model complexity are essential.
• Improved imaging tools are needed for long-term high-resolution imaging over time.

## Introduction

Three-dimensional (3D) cell culture technology is growing by leaps and bounds. The development of 3D *in vitro* models has resulted in the generation of novel platforms, in which one or more physiological aspects of a living organ can be emulated in a controlled manner. Such 3D systems, also known as microphysiological systems (MPS), organ-on-chip (OoC), or tissue-chip, have attracted the attention of the medical and pharmaceutical industries due to their potential to yield new approaches in regenerative medicine and new tools for drug development and testing [1].

Several existing systems incorporate 3D scaffolds that help mimic the natural tissue architecture *in vitro*. These scaffolds are commonly produced by 3D printing, thereby ensuring an accurate deposition of the relevant biomaterials, in an automated fashion. The printing process can readily simulate cellular organization in tissues, *e.g.* by alternating deposition of hydrogels and cells in suspension. This technology is considered to be cutting-edge tech and has a great prospect toward creating custom models. The first research specifically focused on 3D patterning of *in vitro* systems using predesigned scaffolds was reported in the present century, and was driven by their promising biomedical applications [2]. During recent years, advanced 3D-printing techniques, together with a variety of purposely fabricated inks and biocompatible polymers, have demonstrated the enormous potential of this technology for recreating physiological aspects *in vitro*, with excellent reproducibility and high resolution. For example, Edelbrock et al. nicely showed how 3D printing could be used to emulate the characteristic layered structures of cortical brain, using so-called superstructure inks [3].

At present, the most versatile printing procedure is arguably extrusion-based printing, since it is highly controllable, can be adapted to a wide variety of materials, and permits the incorporation of a coaxial nozzle to print sacrificial materials. A notable example was provided by Lee et al., who employed extrusion printing of collagen, modulated by pH-driven gelation, to resolve vascular structures at the micrometer scale [4]. Moreover, the 3D patterning approach proved useful toward producing *in vitro* models with extraordinary precision when employing biomaterials that can support the growth of human-derived cells. In this context, it is worth mentioning the sophisticated work by Lewis and colleagues, who demonstrated the growth and survival of different types of stem cells by creating multiple materials and bio-inks, with controlled composition and structural properties [5].

The potential uses of 3D models with highly characterized scaffolds are manifold and range from the precise *in vitro* modeling of diseases for research purposes to the production of reliable systems for safety and toxicity studies. More exciting are the potential applications to personalized medicine and tissue regeneration, in conjunction with stem cell technology. One of the greatest advantages compared to bare 3D cell cultures or non-printed models is the ability to accurately replicate the architecture and composition of the native tissue. Deposition in 3D also enables rapid, programmable printing of multiple cell types at the same time, thereby creating a highly complex microenvironment [6]. The physiological relevance of 3D models is largely improved when the latter approach is combined with integrated microfluidic technology.

## Challenges

Notwithstanding, several challenges are still to be addressed. The 3D-printing technology gradually improves through multidisciplinary strategies, but additional improvements should be considered to produce more reliable models, and facilitate their translation to the clinical and pharmaceutical industry. The pertinent needs to be considered for the use of 3D-printed scaffolds into physiologically relevant models are mainly related to the biological, biomaterial, and technical components, as well as other factors such as the diffusion of gases/nutrients and complexity reduction [7].

It is thus important to define the application of 3D technologies because the specific strategy will depend on it. In other words, the final objective of the 3D model will condition the prioritization of the biological module over the printing strategy and the system's throughput, or *vice versa*. Once the purpose of the MPS has been defined, the biological and technical aspects should be customized and correctly assembled according to the final goal. The goal might be drug screening, *in vitro* modeling of a disorder, or the design of synthetic implants, among others. Importantly, the biological component must reflect the cell architecture of the organ or tissue to be mimicked, so the choice of cell source is critical. Ideally, phenotypically characterized human-derived cells should be selected to maximize physiological significance. These cell lines offer an alternative to animal-derived models and the possibility of closely simulating molecular processes *in vitro*. Moreover, with the development of stem cell technology, somatic cells from patients can be transformed into stem cells and induce their differentiation to the required cell type. Such reprogrammed cells are known as induced pluripotent stem cells (iPSCs). The generated patient-derived cells containing specific mutations can be integrated or even co-cultured with other cell types to improve the pathological simulation. The latter is always an advantage, but has the complexity of media standardization for all cell types.

Another crucial element is the biomaterial used to support the 3D model. In an *in vitro* system comprising a printed scaffold, the biomaterial component creates the extracellular matrix (ECM) that supports cellular growth and migration within the cell culture. The ECM may consist of natural, synthetic hydrogels or a combination of both. These hydrogels can be polymerized by physical stimuli, such as temperature and UV light, chemical stimuli, such as pH and ionic strength, or biological stimuli (enzymes). Recent studies employ decellularized matrices (dECM) that preserve the natural and mechanical properties of the tissue. The challenge for these hydrogels is fitting the composition and polymerization route to the 3D printing technique, so as to precisely represent the target tissue microarchitecture. Nonetheless, natural or synthetic materials should be characterized in terms of stiffness, rheology, and chemical properties, to generate a reliable bioink. Compatibility of the hydrogel with the selected cell type must be ensured, so additional hallmarks must be met. For example, cellular adhesiveness should be studied to ensure that cells will grow on the scaffold. Porosity, non-cytotoxicity, and degradability are crucial properties to be tailored during the development of stable biological 3D systems.

On the other hand, the technical/instrumental component must resolve the read-out employed for interrogating the cells, so the development of additional devices may be considered to constrain the 3D cell culture or to increase the throughput. Monitoring 3D samples is pretty complex, as compared to the classical growth of cells in monolayers. The currently most suitable techniques to study 3D models are based on fluorescence microscopy or other molecular technologies. The latter however may require retrieval of cells by denaturing the ECM. Raman scattering-based techniques are gradually emerging to compete with their fluorescence-based counterparts [8]. Another non-destructive method for imaging of complex 3D architectures is synchrotron-based micro-CT [9]. An ideal strategy here may involve a multi-parametric assay that allows studying the model from different perspectives and in a non-invasive manner, over multiple time points. The technical approach might also contemplate the integration of mechanical/chemical actuators or remote stimuli to study the cellular response.

The diffusion of nutrients and oxygen is another limitation to bear in mind. The continuous nurturing of cells, as well as the possibility to remove waste and carbon dioxide, are necessary for the long-term survival of the 3D culture. To guarantee this molecular exchange with the culture media, most of the current systems rely on passive diffusion using highly permeable hydrogels. Others combine microfluidic components that simulate the blood flow and ensure an automatic refreshment of the media. However, more sophisticated printed models have integrated a vascularization system within the 3D cell culture, by combining sacrificial printing with microfluidics [9]. This approach is so far, the most advanced method and closely simulates the vascular system of the native tissue.

A different implementation to be considered is the reduction of complexity for the produced 3D model. A simplified fabrication would be ideal to promote high throughput and scalability of the MPS. This is most important for models aiming to perform screening or multiple testing, since generation of multiple data points would be straightforward [10]. Once the model is sustained over time and simplified, validation with sufficient experiments for statistical significance will help identify its intrinsic variations. Comparison of collected data with classical 2D systems or 3D models without a printed scaffold will prove the performance of the new model and represent a step forward to more elaborated designs with higher physiological content.

## Conclusions

**Fig. 1:**
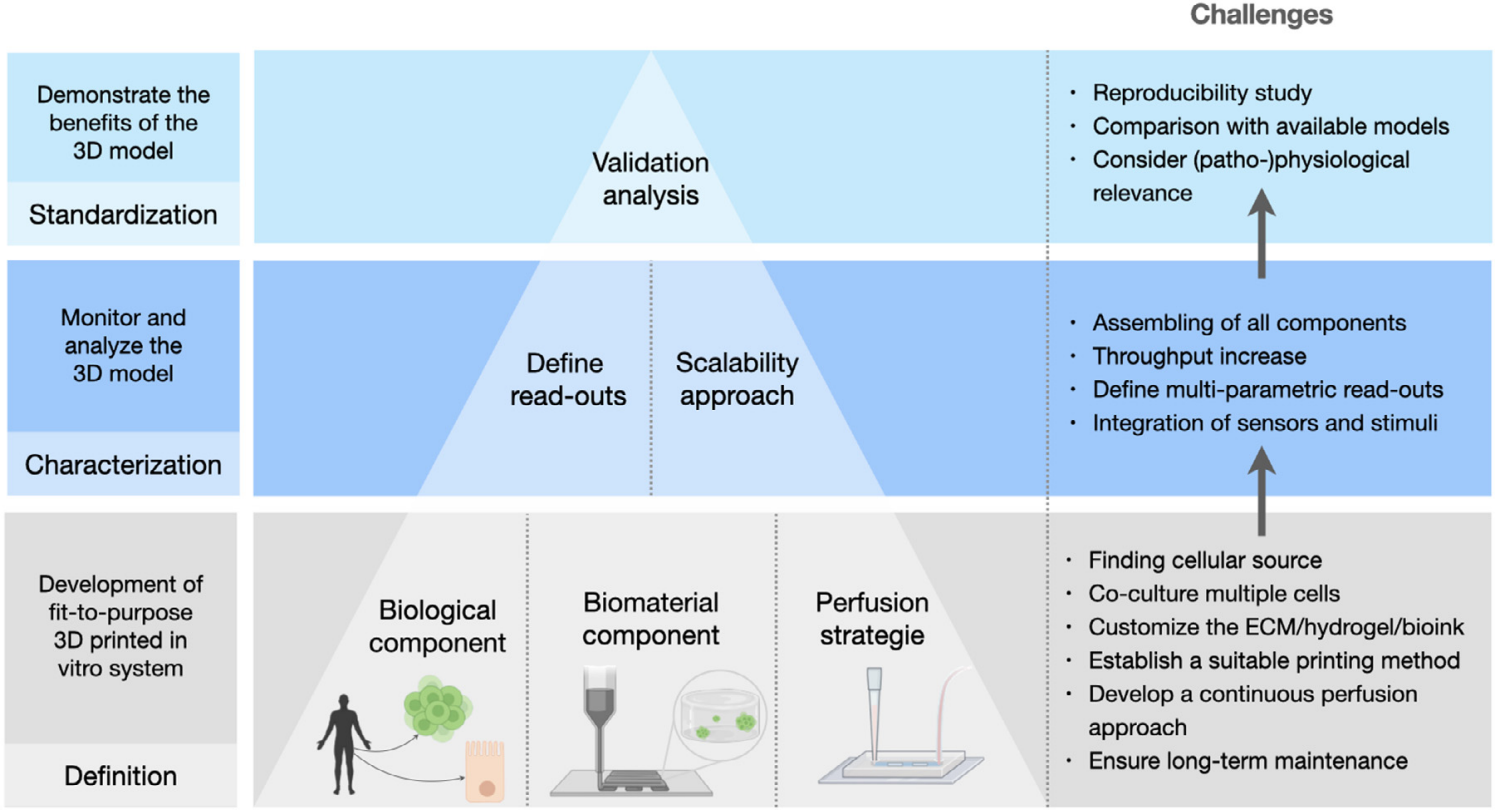
Building blocks for *in vitro* modeling with 3D-printed scaffolds. The scheme summarizes the different factors, from more basic questions to more technical aspects to be considered prior to developing an *in vitro* model involving 3D-printed scaffolds.

The path toward the development of *in vitro* models with 3D-printed technologies requires consideration of multiple factors that will surely improve the accurate examination of cell cultures in 3D, with high reproducibility and reliability (Fig. 1). This can only be achieved by combination of suitable printed scaffolds, stable cell growth, constant perfusion techniques, and reduction of 3D model complexity, thereby sustaining the long-term stability of the system. Overcoming these challenges will ensure the adoption of 3D-printed models for regenerative medicine and for detecting specific toxicity and efficacy outcomes in preclinical studies, not discerned by classical 2D systems.

## Declaration of Competing Interests

The authors declare the following financial interests/personal relationships which may be considered as potential competing interests:

Luis Liz Marzan reports financial support was provided by European Research Council.
